# Primary Leiomyosarcoma of the Colon: A Report of Two Cases, Review of the Literature, and Association with Immunosuppression for IBD and Rheumatoid Arthritis

**DOI:** 10.1155/2018/6824643

**Published:** 2018-01-30

**Authors:** Jessica S. Crystal, Kristin Korderas, David Schwartzberg, Steven C. Tizio, Min Zheng, Glenn Parker

**Affiliations:** ^1^Rutgers Robert Wood Johnson Medical School, New Brunswick, NJ, USA; ^2^Monmouth Medical Center, Long Branch, NJ, USA; ^3^Jersey Shore University Medical Center, Neptune, NJ, USA

## Abstract

Primary leiomyosarcomas (LMS) of the colon are rare and aggressive neoplasms and have been infrequently reported in the literature. These tumors are more aggressive and have poorer prognoses than adenocarcinoma of the colon and are often mistaken as such on initial evaluation. While the former has a clear association with inflammatory bowel disease (IBD), this correlation is not known to exist with LMS and IBD. Nor is there a known link between LMS and the immunosuppression for IBD, despite the known association between malignancy and immunosuppression for other diseases. Due to the low prevalence of this disease entity, there is limited knowledge and literature on the approach to diagnosing and treating these neoplasms, especially in the setting of the aforementioned comorbidities. Here, we describe two cases of this rare entity, presenting in two different circumstances: one in the setting of immunosuppression for IBD and arthritis, with a synchronous urothelial carcinoma, and the second appearing as the source of an acute abdomen. Both diagnoses were established following pathologic analysis.

## 1. Introduction

Primary mesenchymal sarcomas of the gastrointestinal tract are rare and constitute only 0.1–3% of all gastrointestinal tumors [1]. Leiomyosarcoma (LMS) is the most common variant of these tumors and represents only 0.12% of all colon malignancies [2]. Amongst the variety of soft tissue sarcomas, leiomyosarcomas represent 10–20% of these malignancies. LMS most commonly originates in the uterus, GI tract, and retroperitoneum. Within the GI tract, the stomach is the most common site followed by the small intestine, colon, and rectum [3]. LMS of the colon has such a low prevalence that there is a paucity of data describing the true demographic, clinical, or gross features of the disease. We herein report 2 cases of primary LMS of the colon, each of which presented in unusual settings and review the literature to highlight the diagnosis, treatment, association with other diseases, and prognosis of these uncommon malignancies.

## 2. Case Presentations

### 2.1. Case 1

**Figure 1:**
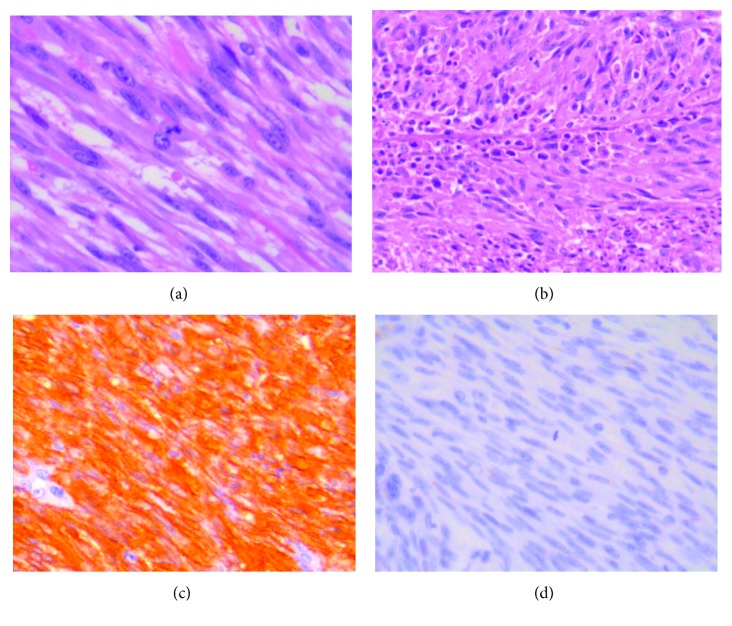
Leiomyosarcoma of the colon. (a) Fascicular tumor cell growth with high mitotic rate (H&E stain, original magnification ×630). (b) Tumor cell necrosis (H&E stain, original magnification ×400). (c) SMA staining positive (×400). (d) c-kit/CD117 staining negative (×400).

**Figure 2:**
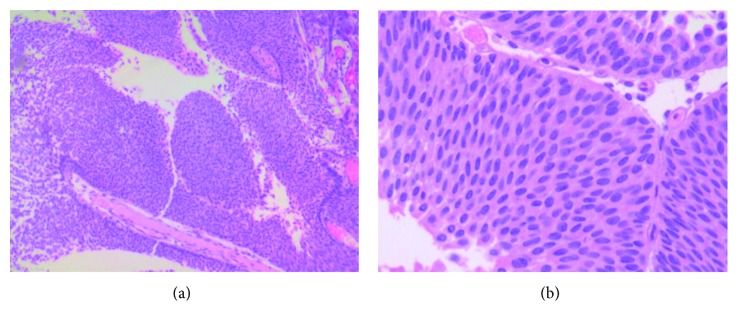
High-grade noninvasive urothelial carcinoma. H&E stain, original magnification: (a) ×100 and (b) ×400.

A 57-year-old female with a past medical history significant for Crohn's disease and arthritis, currently being treated with Humira (adalimumab, AbbVie Inc., North Chicago, IL) and Lialda (mesalamine, Shire US Inc., Lexington, MA), presented for a screening colonoscopy and was found to have a friable mass 40 cm proximal to the anal verge. Initial pathology was consistent with a spindle cell tumor. Further imaging showed extension of the mass towards the bladder. At the time, the patient denied any symptoms of abdominal pain or change in bowel habits. On physical exam, she had a benign abdomen and no other significant findings. Pre-op lab values were within normal limits. The patient then underwent an elective, rectosigmoid colon resection with primary anastomosis, cystoscopy, and transurethral resection of the bladder tumor. Immunosuppression was held for the procedure. Final pathology showed a well circumscribed, pedunculated, firm rectosigmoid colon mass with slightly friable/ulcerated surface. The mass was found to be of high grade (mitotic count up to 40 per 50 high power fields) and showed high Ki67/MIB1 labeling index of up to 70% and stained positive for smooth muscle actin (SMA). The tumor cells were negative for c-KIT/CD117, DOG-1, pankeratin, CD34, and S100 stains (Figure 1). The bladder mass was a tan, pale-pink lesion found to be a high-grade papillary, urothelial carcinoma without invasion into the muscularis propria and no lymphovascular invasion (Figure 2). The patient's postoperative course was complicated by an intra-abdominal abscess which was percutaneously drained and resolved shortly thereafter. She then was discharged home to follow-up for systemic therapy.

### 2.2. Case 2

**Figure 3:**
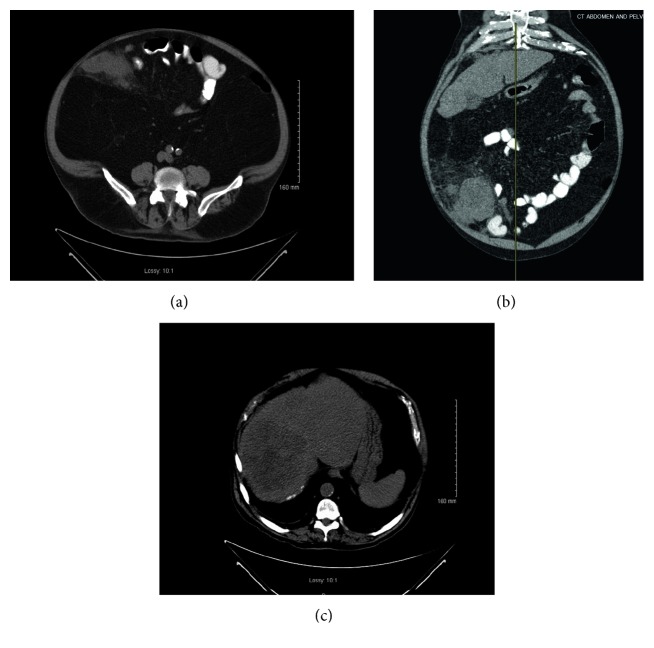
CT scan of the abdomen and pelvis. (a) Axial view of the large tumor in the cecum. (b) Coronal view of the tumor with surrounding inflammatory changes. (c) Large lesion in the right lobe of the liver also seen on CT scan suggestive of metastatic disease.

An 88-year-old male presented to the emergency room with complaints of abdominal pain, fever, and chills. He had an extensive past medical history including atrial fibrillation, obesity, hypertension, chronic kidney disease, hyperlipidemia, left ventricular hypertrophy, benign prostatic hyperplasia, and aortic and mitral valve disease. Abdominal/pelvic computed tomography (CT) revealed an 8.3 × 9.5 × 8 cm cecal mass with thickening of the ascending colon and pericolonic fat stranding. There was no evidence of pneumoperitoneum. There were also several masses in the liver suggestive of metastatic disease, the largest of which measured 12.7 × 11.7 cm (Figure 3). The patient was febrile to 101.5°F and in rapid atrial fibrillation. On physical exam, a tender palpable mass was appreciated in the right lower quadrant along with peritoneal signs. Laboratory work revealed leukocytosis of 20,800 cells/mL and anemia with a hemoglobin value of 10.8 g/dL. The patient underwent an emergent exploratory laparotomy in the setting of his acute peritonitis. Upon entering the peritoneal cavity, foul-smelling fluid was encountered without feculent contamination. A large, dusky cecal tumor was seen covered in fibrinous exudate without any overt focal perforation. A right hemicolectomy was performed. Gross pathologic analysis showed a 6.5  × 5.5  × 5.5 cm firm, partially circumferential cecal tumor with a lobulated, white-gray surface invading into the terminal ileal wall involving the ileal mucosa and mesenteric adipose tissue. Microscopic evaluation of the tumor revealed high-grade leiomyosarcoma. Immunohistochemistry stains revealed CD117 negativity and smooth muscle actin (SMA) positivity confirming leiomyosarcoma. Final pathology revealed a high-grade (grade 3) T2bN0 leiomyosarcoma with abscess and perforation; 12 lymph nodes were negative for malignancy. The patient had a difficult postoperative course with ventilator-dependent respiratory failure and a prolonged stay in the intensive care unit. Eventually, he was transferred to hospice for palliative care and expired within a few months postoperatively.

## 3. Discussion

Leiomyosarcoma is a rare entity to arise primarily from the colon. It originates from the muscularis propria layer of the bowel. While it has been found throughout the colon, it most commonly originates primarily from the sigmoid and transverse colon [4, 5]. It is important to distinguish the diagnosis of LMS from other GI mesenchymal tumors, particularly GIST, as the two diseases have different treatments and prognoses [3]. The introduction of targeted therapies for GISTs contributed to the establishment of further criteria for describing leiomyosarcomas as a separate condition from GIST. Prior to identification of GIST, mesenchymal tumors had been labeled as leiomyosarcomas but were later found to be GISTs [4]. The hallmark histochemical stain that differentiates LMS from GIST is KIT, which is uniformly positive in GIST but are generally negative in LMS. In addition, LMS will stain positive for smooth muscle actin and desmin and will have negative CD117, CD34, and DOG1.1 stains which are positive in GIST [2]. In general, the pathologic analysis of LMS has shown that, like other sarcomas, increased size, mitotic activity, and presence of necrosis confer a poorer prognosis; however, according to case reports, primary colonic LMSs are highly aggressive regardless of size or mitotic activity [1, 6].

The typical presentation of leiomyosarcoma of the gastrointestinal tract is in middle-aged patients with a mean age of diagnosis of 50 years of age [1]. Initial symptoms include abdominal pain, rectal bleeding, intra-abdominal bleeding, weight loss, constipation, diarrhea, bowel obstruction, tenesmus, or fever, which are nonspecific, generally reflect the tumor size and location, and can mimic those symptoms of colonic adenocarcinoma or other gastrointestinal diseases [1, 2]. LMS of the colon was found to be more aggressive compared to other colonic tumors and has a high local recurrence rate and significant hematogenous spread, and rarely lymph node involvement [1, 5]. These tumors are most commonly diagnosed on biopsy obtained during colonoscopy. There has not been any established correlation with any specific tumor markers [2]. When present, metastases most commonly occur in the lungs and peritoneum but can occur in the liver as well. The most common cause of death in these patients is secondary to spread of disease to the lung and liver [5].

Due to the paucity of data, the prognostic factors have been poorly defined and vary by studies. Amongst those identified, poor prognostic factors include age greater than 45 years, necrotic areas within the tumor, dissemination of disease, and tumor size [1, 7]. Accurately predicting patient outcome is difficult due to the rarity of the disease and the short survival once diagnosis has been established. In a recent review of 11 patients with colonic leiomyosarcoma by Aggarwal et al. in 2012, only 2 of the 11 patients survived 5 years, with an average survival of twenty months [6]. Yamamoto et al. reported an estimated 5-year tumor-specific overall survival rate of 51.6% [7].

While the more commonly prevalent colon malignancies, particularly adenocarcinoma, have an increased incidence in patients with inflammatory bowel disease (IBD), sarcomas arising in this setting are quite rare. To our knowledge and by review of PubMed literature, only eight cases of associated sarcoma and IBD have been reported, including three cases of LMS in ulcerative colitis and five cases of sarcomas found in the setting of Crohn's disease [3, 8]. There is however a relationship between malignancy and immunosuppressed patients. Commonly, these patients develop lymphomas or skin cancer; however, recently, there have been a few reports of leiomyosarcoma and other Epstein–Barr virus (EBV) associated smooth muscle tumors arising in younger patients with human immunodeficiency virus (HIV) and those that have been immunosuppressed after receiving transplanted organs [6, 9]. However, this association has not been shown for the immunosuppression that is given to patients with IBD [9, 10]. It is believed that these neoplasms arise in this setting following the complex interaction of several factors. These include the presence of impaired immune surveillance, the role immunosuppressive drugs play in malignancies, a persistently activated lymphoreticular system in response to foreign allograft antigens, and the vulnerability of immunosuppressed patients to viral infections, including ones with malignant potential, such as EBV. It is possible that the poor prognosis of these immunosuppressed patients with tumors may be related to delays in diagnosis in these patients and to the exceptionally aggressive behavior of smooth muscle tumors in the setting of their damaged immune systems [11]. In addition, few cases of urothelial carcinoma of the bladder have been reported in the setting of synchronous leiomyosarcoma of the same organ or presenting simultaneously in 2 different organs [12]. Similarly as rare is the existence of urothelial carcinoma in the setting of IBD [13].

Currently, the best treatment for leiomyosarcoma of the colon is surgical resection. This is true for both those LMS cases that arise independently and those that occur in the setting of immunosuppression and/or IBD [3, 5]. However, since these malignancies are often not detected until late, outcomes are poor, and there is only a 50–60% success rate. Adjuvant chemotherapy is also used, is typically anthracycline or docetaxel based, and does not significantly improve survival [6]. Radiotherapy has not been shown to be as effective [1].

## 4. Conclusion

In conclusion, leiomyosarcoma of the colon is a rare and aggressive neoplasm with poor prognosis. It can mimic other tumors and GI diseases but should be distinguished as a separate entity as the prognosis and treatment vary. While rare, it should be included on the differential diagnoses when a patient presents with a abdominal pain.
